# Monochorionic Diamniotic Twins with Bordering Cord Insertions: Images and Outcome

**DOI:** 10.3390/diagnostics12040985

**Published:** 2022-04-14

**Authors:** Lucian G. Pop, Madalina Piron, Viorica Radoi, Nicolae Bacalbasa, Oana D. Toader

**Affiliations:** 1 National Institute of Mother and Child Care Alessandrescu-Rusescu, 020395 Bucharest, Romania; dumitrascumadalinap@gmail.com (M.P.); vioica.radoi@yahoo.com (V.R.); oana.toader@yahoo.com (O.D.T.); 2 Department of Obstetrics and Gynecology, University of Medicine and Pharmacy Carol Davila, 050474 Bucharest, Romania; nicolae_bacalbasa@yahoo.com; 3 Department of Genetics, University of Medicine and Pharmacy Carol Davila, 050474 Bucharest, Romania

**Keywords:** twins, TTTS, umbilical cord insertions

## Abstract

Twin pregnancy contributes to perinatal mortality, particularly monochorionic diamniotic twin pregnancy. Placental abnormalities are much more common in twin pregnancies than in singletons. In MCDA pregnancy, vascular anastomoses are always present and are accountable for severe complications such as Twin-to-Twin transfusion syndrome (TTTS). In TTTS, umbilical cords are usually inserted at a distance from each other. We present a rare type of MCDA pregnancy, TTTS gr 1 case with bordering umbilical cord insertions.

**Figure 1:**
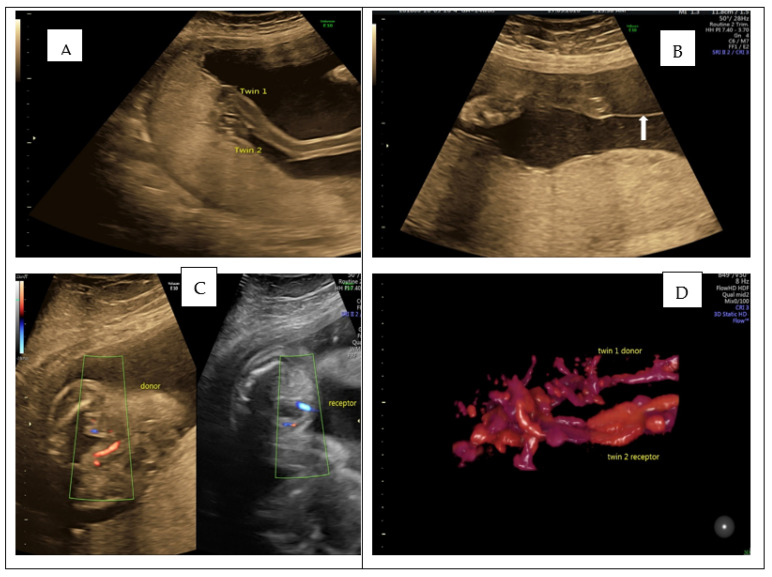
We present the case of a 33-year-old lady, 24 weeks G1P0, referred to our hospital, having been diagnosed with MCDA twin pregnancy and TTTS grade. A viable MCDA pregnancy with TTTS grade 1 was confirmed. Placental cord insertions were extremely close, almost touching one another (**A**). (**B**) White arrow shows a thin amniotic membrane, with bladder visible in both twins in (**C**). A 3D Power Doppler Rendering image, depicting both twins’ close umbilical cord insertion, is shown in (**D**). Captured using Convex Probe 2D-4DRAB6D Voluson E10BT16 (GE Zipfer, Austria). According to ISUOG guidelines, conservative management and laser treatment are sensible options for TTTS gr 1 [1]. We decided to proceed with conservative management, considering cord insertions location, as laser surgery could lead to a catastrophic haemorrhage. The patient was counselled accordingly, and a follow-up plan was set up with a fortnightly assessment. At 33 weeks of gestation, she was admitted to the labour ward with PPROM (preterm prelabour rupture of membrane). She delivered through C section two male foetuses of 1.8 Kg and 2.1 Kg. The babies are now three years of age and thriving.

## Data Availability

Not applicable.
